# A novel therapeutic approach for anaplastic thyroid cancer through inhibition of LAT1

**DOI:** 10.1038/s41598-019-51144-6

**Published:** 2019-10-10

**Authors:** Keisuke Enomoto, Fuyuki Sato, Shunji Tamagawa, Mehmet Gunduz, Naoyoshi Onoda, Shinya Uchino, Yasuteru Muragaki, Muneki Hotomi

**Affiliations:** 1 Departments of Otolaryngology-Head and Neck Surgery, Wakayama Medical University, Wakayama, Japan; 2 Departments of Pathology, Wakayama Medical University, Wakayama, Japan; 3 Department of Breast & Endocrine Surgery, Graduate School of Medicine, Osaka City University, Osaka, Japan; 4 Noguchi Thyroid Clinic and Hospital Foundation, Oita, Japan

**Keywords:** Thyroid cancer, Molecular medicine, Diagnostic markers

## Abstract

A novel therapeutic approach is urgently needed for patients with anaplastic thyroid cancer (ATC) due to its fatal and rapid progress. We recently reported that ATC highly expressed MYC protein and blocking of MYC through its selective inhibitor, JQ1, decreased ATC growth and improved survival in preclinical models. One of the important roles of MYC is regulation of L-neutral amino acid transporter 1 (LAT1) protein and inhibition of LAT1 would provide similar anti-tumor effect. We first identified that while the human ATC expresses LAT1 protein, it is little or not detected in non-cancerous thyroidal tissue, further supporting LAT1 as a good target. Then we evaluated the efficacy of JPH203, a LAT1 inhibitor, against ATC by using the *in vitro* cell-based studies and *in vivo* xenograft model bearing human ATC cells. JPH203 markedly inhibited proliferation of three ATC cell lines through suppression of mTOR signals and blocked cell cycle progression from the G0/G1 phase to the S phase. The tumor growth inhibition and decrease in size by JPH203 via inhibition of mTOR signaling and G0/G1 cell cycle associated proteins were further confirmed in xenograft models. These preclinical findings suggest that LAT1 inhibitors are strong candidates to control ATC, for which current treatment options are highly limited.

## Introduction

Anaplastic thyroid cancer (ATC) has aggressive characteristics such as rapid tumor growth, local invasion, and frequent distant metastasis as compared to differentiated thyroid cancers (DTC) like papillary and follicular thyroid cancer, and it is one of the most fatal cancers in human. Generally, DTC have a good survival ratio that was reported over 90% at 10 years after surgery^1–5^. Though ATC is treated with a multidisciplinary approach including surgery, radiotherapy and chemotherapy, the survival ratio of ATC was only 36% at six months, and 18% at 1 year^6^.

Recently, the molecular targeted therapies have been developed for ATC^7,8^. Of them, lenvatinib successfully prolonged progression free survival not only in DTC but also in ATC^9,10^. This molecular targeted therapy, which has been approved clinically in Japan, inhibits receptor tyrosine kinase (RTK) pathways and sometimes has severe complications such as uncontrollable hypertension, proteinuria, aerodigestive fistula and fatal bleeding from tumor site^10,11^. Moreover, ATC may develop resistance to these RTK inhibitors after using them for a longtime. Therefore, novel targeted therapies are urgently needed for ATC.

The L-neutral amino acid transporter 1 (LAT1) protein located in cell surface membrane constitutes heterodimer with 4F2hc (LAT1/4F2hc complex), asymmetrically. It transports the essential amino acids such as methionine, isoleucine, and phenylalanine^12^. Many malignant tumors overexpress LAT1, and the poor overall survival rate was shown in LAT1 expressed cases of major cancers such as glioma^13^, tongue cancer^14^, laryngeal cancer^15^, hypopharyngeal cancer^16^, breast cancer^17^, esophageal cancer^18^, non-small cell lung cancer^19^, pancreatic cancer^20^, hepatocellular carcinoma^21^, ovarian cancer^22^ and so on. LAT1 promoter has a canonical MYC binding sequence and overexpression of MYC increased LAT1 promoter activity^23^. Additionally, the knockdown of MYC leads to reduction of LAT1 expression in cancer cell lines^23^. Another study showed that MYC selectively upregulates LAT1 and LAT3 expression, and promotes LAT1 and LAT3 mediated essential amino acid uptake^24^. Enomoto and Zhu *et al*. have recently shown that the MYC transcription program has been activated in ATC, and the MYC inhibition by JQ1, a bromodomain inhibitor, is particularly sensitive to proliferation and tumor invasion^25,26^. Therefore, we anticipate that ATC, in which MYC expression was activated, would also elevate LAT1 expression, and then the inhibition LAT1 signaling may lead to tumor regression in ATC.

LAT1 inhibitor, JPH203, is one of the most promising potential novel drugs targeting an amino acid transporter in breast, gastric, pancreatic, and prostate cancers with LAT1 amplification, and it has already been underway for clinical phase 1 and 2 trials for some of the advanced solid tumors. Here, we report that LAT1 and co-expressed 4F2hc have been upregulated in ATC. Moreover, we showed that JPH203 was effective in suppressing tumor proliferation via mTOR signaling by cell-based studies and a mouse xenograft model.

## Materials and Methods

### Histological analysis

**Figure 1:**
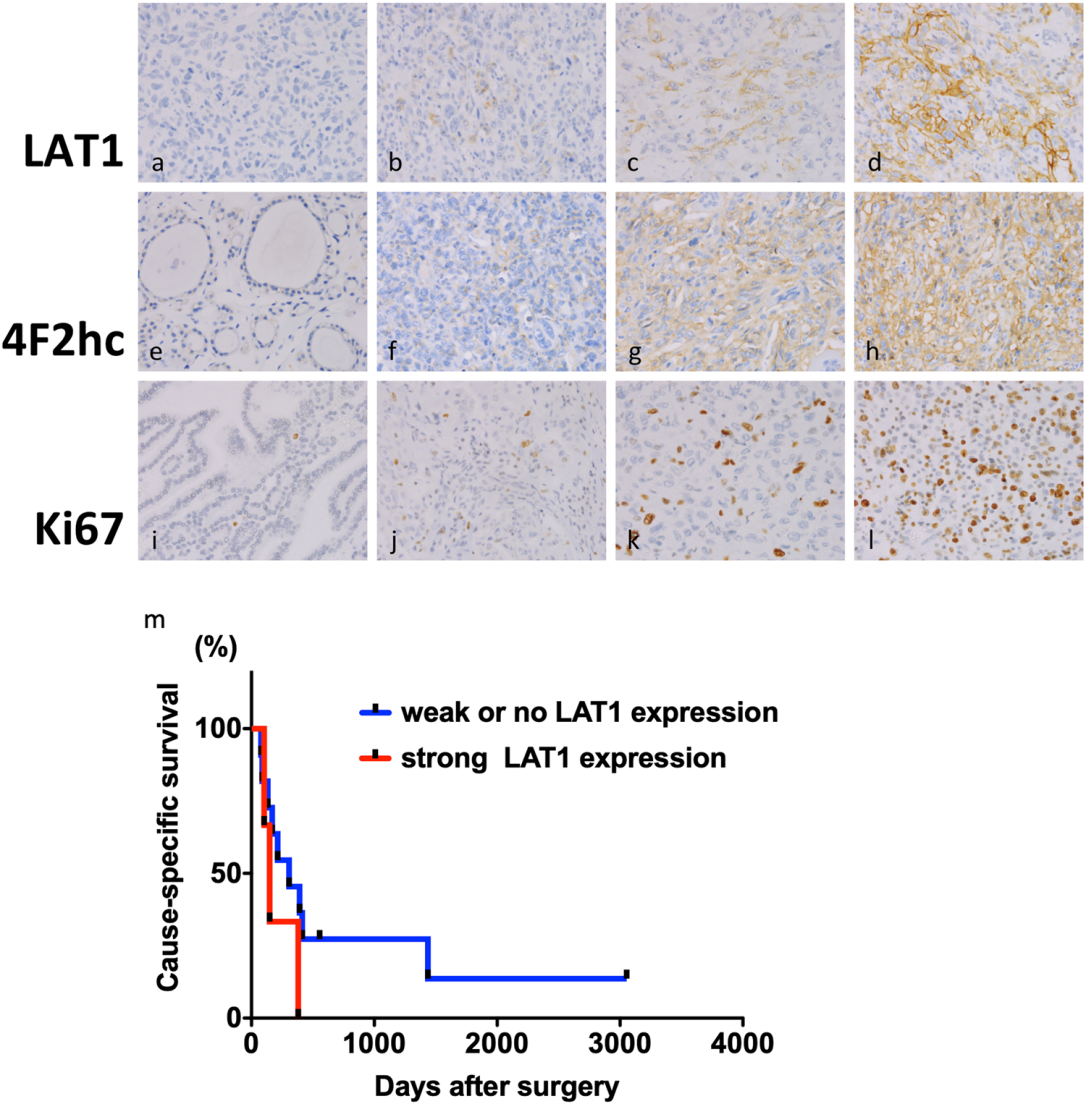
Representative images of immunoreactivity for LAT1, 4F2hc, and Ki67 in human ATC and AG tissues. (**a**) immunoreactivity 0 for LAT1 in ATC tissues; (**b**) immunoreactivity 1 for LAT1 in ATC tissues; (**c**) immunoreactivity 2 for LAT1 in ATC tissues; (**d**) immunoreactivity 3 for LAT1 in ATC tissues. (**e**) immunoreactivity 0 for 4F2hc in AG tissues; (**f**) immunoreactivity 1 for 4F2hc in AG tissues; (**g**) immunoreactivity 2 for 4F2hc in ATC tissues; (**h**) immunoreactivity 3 for 4F2hc in ATC tissues. (**i**) immunoreactivity 0 for Ki67 in AG tissues; (**j**) immunoreactivity 1 for Ki67 in ATC tissues; (**k**) immunoreactivity 2 for Ki67 in ATC tissues; (**l**) immunoreactivity 3 for Ki67 in ATC tissues. (**m**) Cause-Specific Survival (CSS) rates of ATC patients according to LAT1 expression.

Samples of 14 ATC and 15 non-cancerous thyroid tissues as controls were obtained from the Noguchi Thyroid Clinic and Hospital Foundation, Beppu, Japan, in accordance with ethics approval from Noguchi Thyroid Clinic and Hospital Foundation (No. 020) and Wakayama Medical University School of Medicine (No. 2449). All research was performed in accordance with relevant guidelines and regulations, and the written informed consent was obtained from all participants. Immunohistochemistry (IHC) was performed using Discovery Auto-Stainer with automated protocols from sections of formalin-fixed paraffin embedded human and mouse blocks (Ventana Medical Systems, Inc., Tucson, AZ; Roche, Mannheim, Germany). Primary antibodies used are anti-LAT1 (1:500 dilution; ab208776), anti–Ki67 (1:300 dilution; ab32072) from Abcam (Cambridge, MA) and anti-4F2hc/CD98 (1:500 dilution; #94274) from Cell Signaling Technology (Boston, MA). Each sample was analyzed by two clinical pathologists (F.S. and Y.M.) who graded staining intensity and distribution in non-cancerous as well as cancer cells. As shown in Fig. 1, the distribution of staining was recorded as 0 (negative staining in the cell membrane), 1 (less than 10% of weak staining in the cell membrane), 2 (more than 10% of weak or moderate staining in the cell membrane) and 3 (more than 50% of strong staining in the cell membrane). The quantitative analysis was carried out for Ki67 immunoreactivity with ImageJ64 software (ImageJ; Wayne Rasband, National Institutes of Health) in xenograft mouse model.

### Survival analysis

The details of the patients’ outcome were obtained from the Noguchi Thyroid Clinic and Hospital Foundation, Beppu, Japan, computerized database, and these data were confirmed using the medical records. Survival rate from the date of surgery until death or loss of contact (Cause-Specific Survival; CSS) was estimated by the Kaplan-Meier method. Statistical significance for survival was compared using the log-rank test.

### Cell culture

The 3 human ATC cells, 8505C, OCUT-2 and OCUT-6, were used in the present study. The 8505C was purchased from the American Type Culture Collection (ATCC). The other two cells (OCUT-2 and OCUT-6) were established by N.O., previously^27^. These three cells have different genetic characteristics that were detailed by Onoda N. *et al*., Ito T. *et al*. and Zhang L. *et al*.^27–29^. In brief, 8505C has p53, BRAF, EGFR and PI3K3R1/2 mutation, OCUT-2 has both BRAF and PI3KCA mutation, and OCUT-6 has NRAS mutation. The cells were cultured in RPMI-1640 media supplemented with 10% fetal bovine serum, 1% sodium pyruvate, and 1% antibiotic-antimycotic solution in 5% CO_2_ at 37 °C degree in humidified incubator. The medium was replaced every 48 hours.

### Western blot

Cultured cells or tumor tissues were washed with phosphate-buffered saline (PBS) and homogenized in a solution with 50 mM Tris buffer, 150 mM NaCl, 1 mM ethylene-diaminetetraacetic acid, 0.1% Triton-X^®^ 100 (Sigma Aldrich, St. Louis, MO), 1% NP40 and proteinase/phosphatase inhibitors. After centrifugation at 16000 rpm for 10 min, lysates were used for western blot analysis. Protein concentration was determined using the Pierce Coomassie Plus Protein Assay Kit (Thermo Fisher Scientific, Cambridge, MA) according to manufacturer’s protocol. Sodium dodecyl sulfate polyacrylamide gel electrophoresis (SDS-PAGE) was then performed on the cell lysates, transferred to polyvinylidene fluoride membranes, and immunostained by iBind Western Device (Thermo Fisher Scientific, Cambridge, MA) using antibodies as described below. The antibodies used were against LAT1 (1:200 dilution; #PA5-50485), cyclin D1 (1:500 dilution; #33-3500) from Thermo Fisher Scientific (Cambridge, MA), 4F2hc/CD98 antibody (1:500 dilution; #94274), CDK4 (1:1000 dilution; #12790), phosphorylated 4EBP1 (T37/46) (1:1000 dilution; #2855), 4EBP1 (1:1000 dilution; #9452), phosphorylated S6 (S235/236) (1:2000 dilution; #4858), S6 (1:1000 dilution; #2217), phosphorylated p70S6K (T389) (1:1000 dilution; #9234), p70S6K (1:1000 dilution; #9202) from Cell Signaling Technology (Boston, MA), E2F-1 (1:200 dilution; sc-251) from Santa Cruz Biotechnology (Dallas, TX), and α-tubulin (1:10000 dilution; ab7291) from Abcam (Cambridge, MA). The horseradish peroxidase-conjugated anti-mouse immunoglobulin G (IgG) (1:1000 dilution; #7076) or anti-rabbit IgG (1:1000 dilution; #7074) from Cell Signaling Technology (Boston, MA) were used as the secondary antibody, and subsequently detected by means of an ECL system (Amersham ECL Prime, GE Healthcare, UK). For control of protein loading, the blots were striped with Re-Blot Plus (Millipore Sigma, Temecula, CA) and re-probed with total form proteins and α-tubulin. The ImageJ64 software was used for quantification of band intensities. Western blot analyses were repeated 2 times, each with triplicates from independent tissues.

### Immunofluorescence staining

8505C, OCUT-2 and OCUT-6 human ATC cells were cultured in the medium with JPH203 at the concentration of 100 μM or DMSO as control. After 24 hours, cells were fixed in culture well with 4% paraformaldehyde for 30 min. Then cells were permeabilized with 0.1% Triton X-100 for 10 min, blocked with 10% donkey serum for 1 hour. Primary antibodies used were anti-LAT1 antibody (1:50 dilution; sc-374232) from Santa Cruz Biotechnology (Dallas, TX), anti-4F2hc/CD98 antibody (1:800 dilution; #13180) from Cell Signaling Technology (Boston, MA), and anti–Ki67 antibody (1:1000 dilution; ab92742) from Abcam (Cambridge, MA). After washing three times with 1 × PBS, cells were incubated for 1 hour with anti-mouse secondary antibodies conjugated to Alexa Fluor^®^ 488 fluorescent dye (1:1000 dilution; #4408) and anti-rabbit secondary antibodies conjugated to Alexa Fluor^®^ 555 fluorescent dye (1:1000 dilution; #4413) purchased from Cell Signaling Technology (Boston, MA). 4, 6-diamidino-2-phenylindole (DAPI) at the concentration of 1 mg/ml was used for nuclear visualization and was added at the end of the process. Images were collected by fluorescence microscopy.

### XTT assay

The Cell Proliferation Kit II (XTT) assay (Sigma Aldrich, cat. 11465015001; St. Louis, MO) was used according to manufacturer’s protocol. Briefly, cells were grown in 96-well microplates in a final volume of 100 μl culture medium per well. The cells were incubated with JPH203 for 24 hours and then 50 μl of the XTT labeling mixture (final XTT concentration 0.3 mg/ml) was added to each well. After incubation of the microplate for 4 hours in 5% CO_2_ at 37 °C in humidified incubator, the formazan dye formed was quantitated using a scanning multi-well spectrophotometer (MTP-450 microplate reader, Corona, Hitachinaka, Japan).

### Cell proliferation assays

To evaluate the effect of JPH203 on cell proliferation, 8505C, OCUT-2, and OCUT-6 cells were cultured in the medium with the increasing concentrations of JPH203 (100 μM) or DMSO as control in 6-well plates in triplicates. Cell counts were measured every 24 hours for 96 hours using a Countess™ automated cell counter under the manufacturer’s protocol (Invitrogen, Carlsbad, CA).

### Flow cytometric analysis of cell cycle

Cells were seeded into six-well plates at a density of 3 × 10^5^ cells. After 24 hours, JPH203 at 100 µM concentration was applied for 24 hours at 37 °C. Control cells were treated with DMSO. After treatment, all harvested cells were washed with ice-cold PBS and then fixed with cold 70% (vol/vol) ethanol and stored at −20 °C for at least 2 hours. After this, cells were washed in PBS buffer and then incubated with 1 mg/mL DAPI solution for 10 minutes before being analyzed. The cell cycle profiles were determined using flow cytometry (LSRFortessa II, BD Bioscience, San Jose, CA) and analyzed with FlowJo (FlowJo LLC, Ashland, OR). All experiments were performed at least triplicate.

### Knockdown of LAT1 by RNA interference

Short interference RNA (siRNA) against LAT1 was purchased from QIAGEN (Cat. No. 1027416; Hilden, Germany). The target sequences of LAT1 were siRNA_#1; TGGGCTTGTGACATTCGTGAA and siRNA_#2; GAACATTGTGCTGGCATTATA, respectively. The negative control siRNA was also obtained from QIAGEN (Cat. No. 1022076; Hilden, Germany). 8505C, OCUT-2, and OCUT-6 cells at a density of 1 × 10^5^ cells in each well were cultured for 16 hours in 6-well plates. The siRNAs were transfected into the cells using the Lipofectamine RNAiMAX reagent (Thermo Fisher Scientific, Cambridge, MA). After transfection, the cells were incubated for 24 hours at 37 °C and subjected to further analyses.

### *In vivo* xenograft tumor assays

All animal experiments were performed under protocols approved by the Animal Care and Use Committee of Wakayama Medical University (No. 877), and all methods involving animals were performed in accordance with the relevant guidelines and regulations. Female athymic nude mice with ages of 6 to 8 weeks old (BALB/c-nu, CAnN.Cg-*Foxn1*^*nu*^/CrlCrlj, Charles River Laboratories Japan, inc., Yokohama, Japan) were used for the xenograft assays. 8505C cells (1 × 10^6^ cells) in 200 μl suspension mixed with Matrigel basement membrane matrix (BD Biosciences, cat. 354234; San Jose, CA) were inoculated subcutaneously into the right flank of mice. After 3 days, the mice were randomly divided into two groups: a control group (n = 5), and JPH203 treated group at 12.5 mg/kg/day concentration (n = 5). JPH203 was first dissolved in DMSO and diluted to 3 volumes of β-cyclodextrin (Sigma Aldrich, cat. H107-5G, St. Louis, MO) in pure water and administered through intraperitoneal injection. The tumor volume was calculated as L x W x H x 0.5236 (mm). JPH203 or vehicle treatment was applied when the median tumor size reached at approximately 100 mm^3^. Tumor growth rate was calculated using the following equation: The tumor growth rate = (V2-V1)/(t2-t1) in which, V2: tumor volume at euthanasia; V1: tumor volume when JPH203 injection was started; t2: the day at euthanasia; t1: the day when JPH203 injection was started.

Treatment was continued until the first mouse reached humane endpoint criteria, upon which all mice were euthanized using isoflurane inhalation. Then, the tumor tissues were collected for further analysis. Formalin-fixed paraffin-embedded sections from the internal organs were analyzed by hematoxylin and eosin (HE) staining according to standard methods. All slides were reviewed by the pathologists, and were photographed using an NIKON Microscope (NIKON, Tokyo, Japan) with standard software.

### Statistical analysis

Data are expressed as mean ± standard error (SE). All tests were two-sided and *p* < 0.05 was considered to be statistically significant. GraphPad Prism version 5.0 for Mac OS X (GraphPad Software, La Jolla, CA) was used to perform analyses of variances.

## Results

### Overexpression of LAT1 and 4F2hc in human ATC tissues

We first investigated the expression of LAT1, 4F2hc and Ki67 in human ATC tissues by IHC. The distribution of each molecules was summarized in Table 1. Significant LAT1 immunoreactivity, at least score 1, was detected in the cancer tissues of ATC (78%: 11/14 cases) as compared with the adjacent non-cancerous tissues and it was predominantly localized in the cell membrane of cancer cells (Fig. 1). On the other hand, no LAT1 immunoreactivity was observed in all adenomatous goiter (AG) tissues (0%: 15/15 cases). 4F2hc immunoreactivity over the score 2 was detected in the all ATC tissues (100%: 14/14 cases), and it was highly detected in the AG tissues as well (67%: 10/15 cases). Ki67 immunoreactivity over the score 2 was frequently shown in the ATC tissues (85%: 12/14 cases), whereas it was barely detected in AG tissues (6%: 1/15 cases).

**Table 1:** Immunohistochemical expression of LAT1, 4F2hc, and Ki67 proteins in human anaplastic thyroid cancer and adenomatous goiter as control.

Case	Score					
	Age	Sex	Histology	LAT1	4F2hc	Ki67
1	57	F	ATC	1	3	3
2	82	M	ATC	1	3	3
3	61	M	ATC	2	2	3
4	64	F	ATC	1	3	3
5	70	F	ATC	1	2	2
6	75	M	ATC	1	3	3
7	57	F	ATC	3	3	3
8	78	F	ATC	0	2	2
9	69	M	ATC	1	2	3
10	56	F	ATC	0	2	1
11	56	M	ATC	1	2	2
12	74	F	ATC	3	3	3
13	69	F	ATC	1	2	2
14	77	F	ATC	0	2	1
15	82	F	AG	0	2	1
16	53	F	AG	0	3	0
17	53	M	AG	0	0	0
18	66	F	AG	0	2	1
19	83	F	AG	0	1	1
20	65	F	AG	0	3	1
21	67	F	AG	0	3	1
22	46	F	AG	0	3	1
23	59	F	AG	0	2	1
24	73	F	AG	0	1	1
25	66	F	AG	0	1	2
26	64	F	AG	0	2	0
27	43	F	AG	0	1	0
28	81	F	AG	0	2	1
29	58	M	AG	0	3	0

ATC, Anaplastic Thyroid Cancer; AG, Adenomatous Goiter as control.

To know whether overexpressed LAT1 is associated with survival outcome or not, we further analyzed the survival data and the score of LAT1 expression (Fig. 1m). The patients with strong LAT1 expression of score 2 or 3, were associated with poor prognosis than those with weak or no LAT1 expression of score 1 or 0, but there was no significance (p = 0.2291). Cause-Specific Survival (CSS) at 1 year were 0 and 45.5%, respectively.

### Human ATC cells express LAT1and 4F2hc proteins

**Figure 2:**
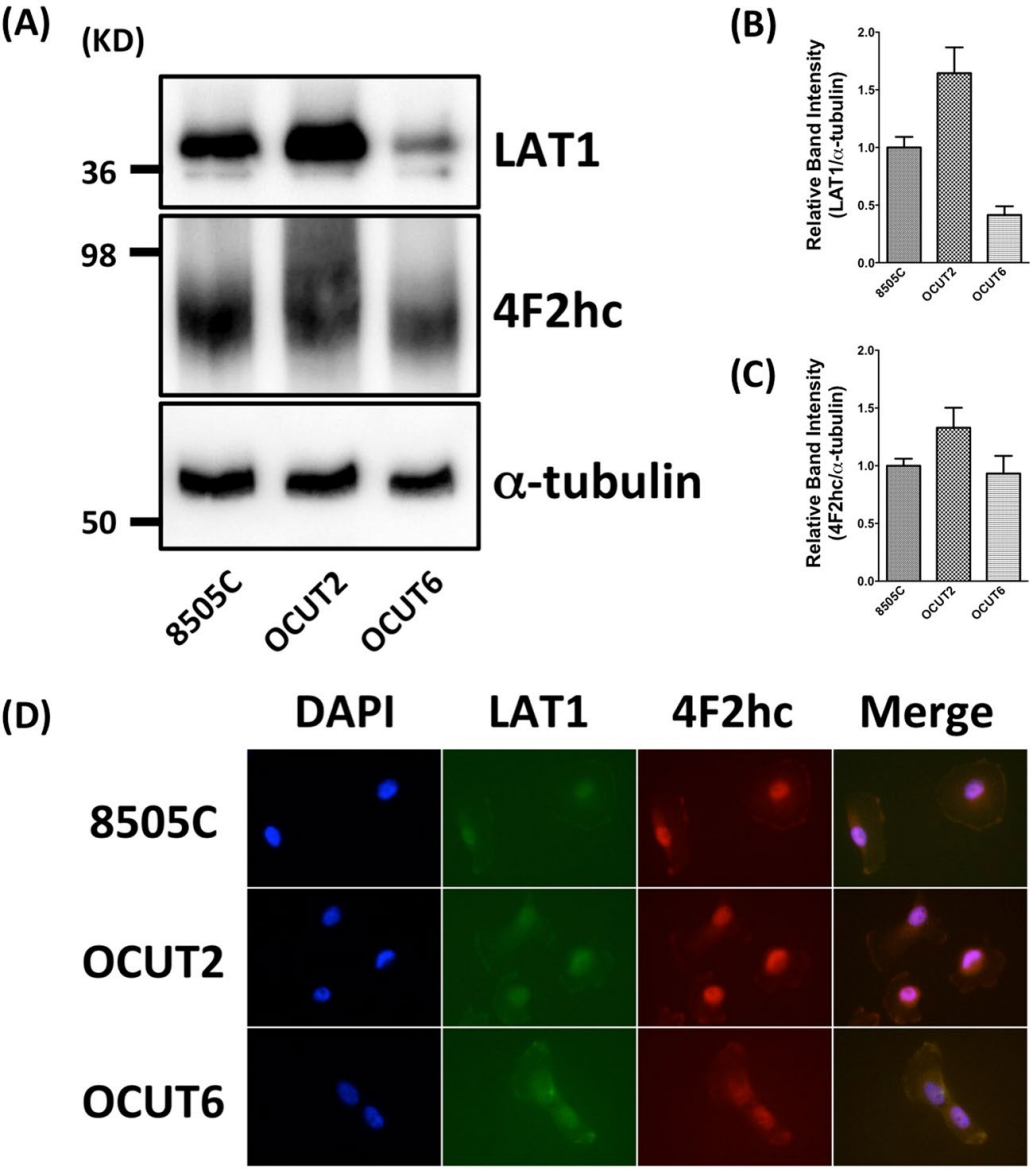
Detection of LAT1 and 4F2hc by western blotting and immunofluorescence analyses in 8505C, OCUT-2, and OCUT-6 human ATC cells. (**A**) Expression of LAT1 and 4F2hc levels in the indicated cells detected by western blotting. (**B**) The densitometry value of LAT1 was normalized α-tubulin. (**C**) The densitometry value of 4F2hc was normalized α-tubulin. (**D**) DAPI (4,6-diamidino-2-phenylindole is a blue fluorescent stain specific for DNA) nuclear staining was shown in blue. Both LAT1 (green) and 4F2hc immunoreactivities (red) were detected on the plasma membrane of 8505C, OCUT-2, or OCUT-6 cells. LAT1 (green) and 4F2hc immunostaining (red), and DAPI (blue) were merged and shown in 2D, indicating the coexistence of LAT1 with 4F2hc in the plasma membrane of 8505C, OCUT-2, or OCUT-6 cells, respectively.

To confirm the expression of LAT1 and 4F2hc in human ATC cells, we first performed western blot analysis for 8505C, OCUT-2, and OCUT-6 cells. All three cells were confirmed for the expressions of LAT1 and 4F2hc proteins (Fig. 2A). We also noticed that the level of LAT1 expression rate was in order in OCUT-2 > 8505C > OCUT-6 (Fig. 2B) and of 4F2hc expression rate was in order in OCUT-2 > 8505C = OCUT-6 (Fig. 2C) according to western blot analysis. Additionally, we confirmed the localization of the LAT1 and 4F2hc proteins by immunocytochemistry. Both LAT1 and 4F2hc antibodies accumulated at the cell membrane (Fig. 2D). These data indicate that these three human ATC cells absolutely express the LAT1 together with 4F2hc.

### JPH203 inhibits cell proliferation in human ATC cells

**Figure 3:**
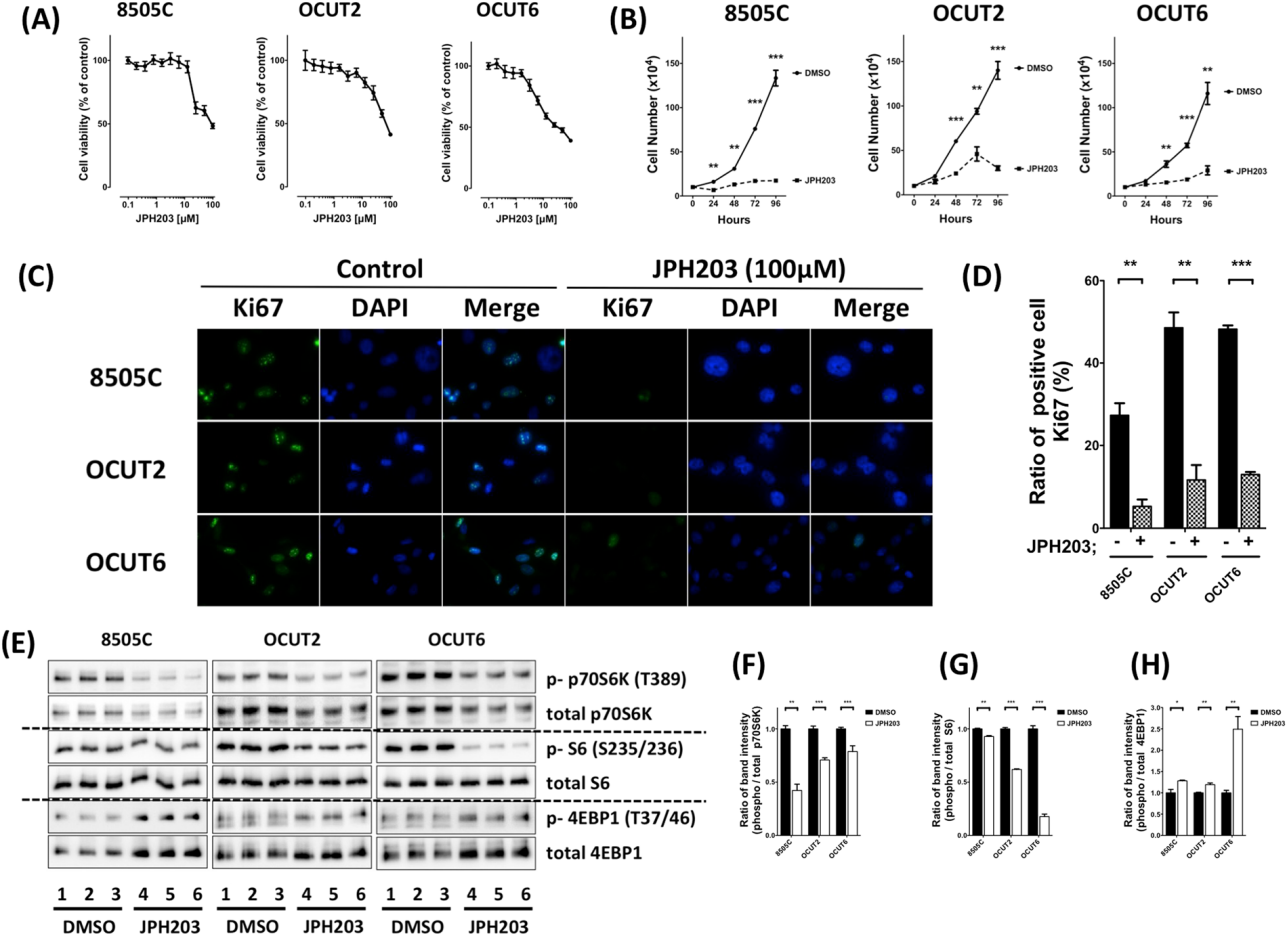
Effect of JPH203 on cell viability (**A**) and tumor cell growth (**B**) in 8505C, OCUT-2 and OCUT-6 cells. (**B**) Three cells were treated with vehicle (DMSO) or JPH203 at increasing doses of 100 µM for 24, 48, 72, or 96 hours. Cells were harvested, and cell numbers were counted. (**C**) Detection of Ki67 (green) by immunofluorescence analyses in 8505C, OCUT-2, and OCUT-6 cells. DAPI nuclear staining was shown in panel (blue). Ki67 and DAPI were merged and shown in right panels. (**D**) Quantification of Ki67-positive cells. After treatment with 100 µM JPH203 for 24 h, 8505C, OCUT-2, and OCUT-6 cells had significantly lower numbers of cells stained for Ki67 than those treated with DMSO as control. (**E**) Western blot showing phosphorylation of p70S6K, S6, and 4EBP1 in 8505C, OCUT-2, or OCUT-6 cells with 100 µM JPH203 for 24 h or DMSO as control (**F**–**H**). The band intensities were quantified and compared in these mTOR signals (*p < 0.05, **p < 0.01, ***p < 0.001).

We further investigated whether a selective LAT1 inhibitor, JPH203, inhibits ATC progression or not. To examine the effect on cell viability, 8505C, OCUT-2 and OCUT-6 cells were treated with JPH203 at various concentrations using an XTT assay. As shown in Fig. 3A, when cells were treated with 0.1 to 100 µM JPH203 for 2 days, it potently inhibited cell proliferation in all human ATC cells in a dose dependent manner. To test whether JPH203 was effective in inhibiting LAT1 in human ATC, we evaluated the effect of JPH203 on the proliferation of these three human ATC cells: 8505C, OCUT-2 and OCUT-6 cells. After 96-hour exposure at the concentration of 100 µM, JPH203 showed inhibition of 87.0%, 78.6%, and 75.0% in proliferation of 8505C, OCUT-2 and OCUT-6 cells, respectively (Fig. 3B). Of note, JPH203 was similarly effective in inhibiting cell proliferation irrespective of the genetic background of these three cell lines. We performed further immunofluorescence staining analysis to assess the extent of JPH203-induced inhibition of tumor cell proliferation using the cell proliferation marker Ki67. Figure 3C compares the number of cells positively stained with Ki67 in human ATC cells. JPH203 treatment significantly decreased the number of Ki67-positive cells as shown in Fig. 3C,D.

Previous reports showed that JPH203 inhibited amino acid transport and suppressed the mTOR signals in other cancer types^30–34^. Thus, the three ATC cells prompted us to examine whether the phosphorylated p70S6K, S6 and 4EBP1 proteins’ abundance, which are the downstream of mTOR, were affected by JPH203 or not. In accordance with the previous reports in other carcinomas, western blot revealed that JPH203 inhibited the mTOR signaling pathway in the human ATC cells as shown in Fig. 3E. The counting data of western blotting showed statistically significant differences in JPH203 group as compared to the control group in all human ATC cells (Fig. 3F–H).

**Figure 4:**
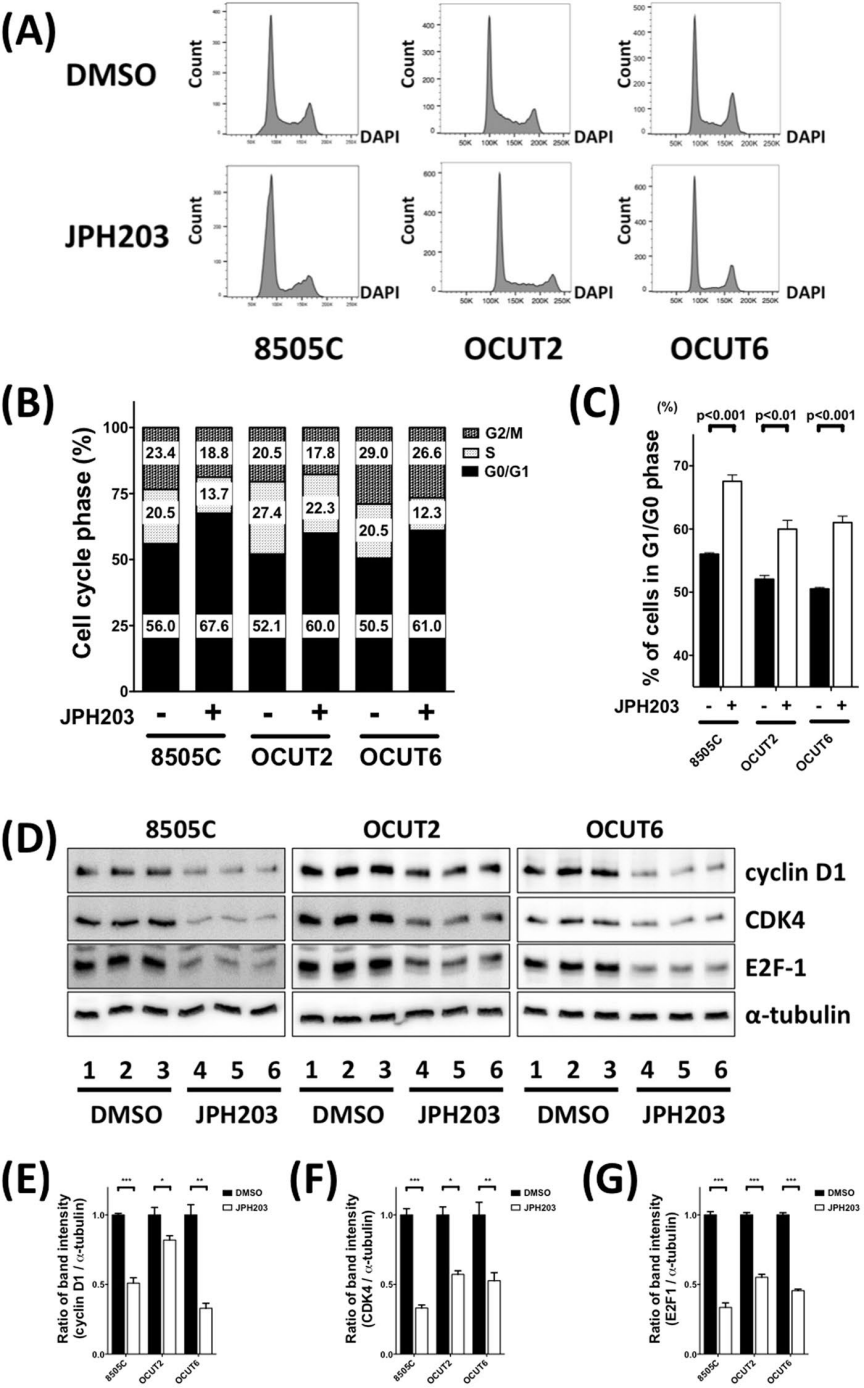
JPH 203 induced cell cycle arrest in human ATC cells. (**A**) Cell cycle analysis after 24 hours of JPH203 treatment. The percentages of cell populations of different cell cycle phases are shown in the upper-right corner. After JPH203 treatments, 8505C, OCUT-2, and OCUT-6 were blocked in the G1/G0 phase, and there was reduction in the S and G2/M phases. (**B**) JPH203 treatment affected the distribution of cell cycle. Different cell cycle phases were quantified by DAPI staining, followed by fluorescence-activated cell sorting analysis in our human ATC cells. (**C**) JPH203 treatment lengthened the G1/G0 phase in our human ATC cells (**p < 0.01, respectively). (**D**) A representative western blot for expression of cell cycle-associated proteins, including cyclin D1, CDK4 and E2F1 in 8505C, OCUT-2, and OCUT-6 cells after treatment with 100 µM JPH203 for 24 h or control DMSO. (**E**–**G**) The band intensities were quantified, and compared after normalize to α-tubulin (*p < 0.05, **p < 0.01, ***p < 0.001).

We therefore examined cell cycle distribution in human ATC cells with or without JPH203 inhibitors. The FACS assay confirmed that treatment with JPH203 increased percentage of G0-G1 phase cells in all human ATC cells (Fig. 4A). Quantified results confirmed the statistically significant increase of G0-G1 phase cells by JPH203 inhibitors (Fig. 4B,C). Consistent with cell cycle distribution findings, western blot assay results also confirmed that cyclin D1, CDK4 and E2F1, the protein associated with G1 restriction checkpoint of cell cycle, were also reduced (Fig. 4D). The counting data of western blot also showed statistically significant differences in JPH203 group as compared to control group in all human ATC cells (Fig. 4E–G).

### LAT1 knockdown inhibits cell proliferation in human ATC cells

To reveal effects of JPH203 as LAT1 knockdown, we further examined whether LAT1 knockdown affected mTOR signaling and subsequent cell proliferation in ATC cells or not. Both siRNA_#1 and _#2 for LAT1 knockdown significantly decreased endogenous LAT1 protein expression in 8505C, OCUT-2, and OCUT-6 (Supplemental Fig. S1). Expressions of 4F2hc and phosphorylated p70S6K were decreased by LAT1 knockdown using siRNA_#1 and _#2. In addition, the cyclin D1 expression was significantly decreased by LAT1 knockdown using each of siRNA_#1 and _#2.

### JPH203 suppressed ATC cell growth in mouse xenograft models

**Figure 5:**
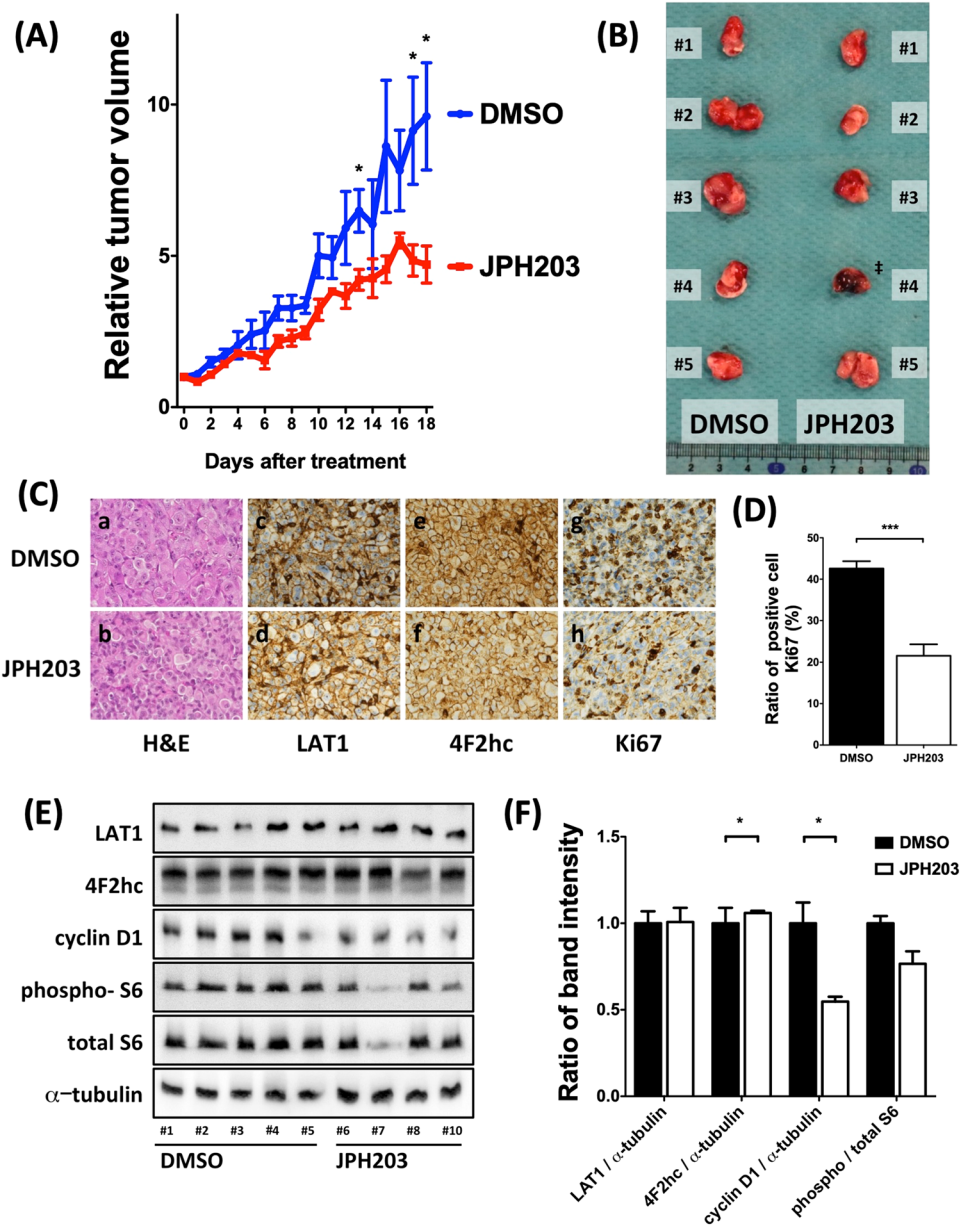
Anti-tumor effect of JPH203 in 8505C-inoculated athymic BALB/c nude mice. An equal number of 8505C cells (1 × 10^6^ cells) were injected into the flanks of each mouse before treatment. When tumors started to develop (average tumor size reached 100 mm^3^), JPH203 or vehicle was administered intraperitoneally for 18 day (12.5 mg/kg/d). JPH203 treatment decreased (**A**) tumor growth ratio, (**B**) tumor size. ‡: #4 mouse was just died before euthanasia. Therefore, this mouse was excluded from following analysis. (**C**) Representative images of H&E-stained tumor sections (panels a and b). There was no difference between vehicle treated mice (panel a) and JPH203 treated mice (panel b). Immunohistochemical analysis showed the LAT1 and 4F2hc express expression at cell surface, and their expressions had little effect on JPH203 treatment (panel c to f). However, JPH203 treatment decreased the Ki67 immunoreactivity (panel g and h). (**D**) The counting data showed the number of Ki67 immunoreactive positive cells were decreased in the group of JPH203 treated mice (***p < 0.001). (**E**) Western blot for LAT1, 4F2hc, cyclin D1, phosphorylated S6, total S6 and α-tubulin was carried out to access mTOR signal and cell cycle regulators. (**F**) The band intensities of these proteins detected in (**E**) were quantified and compared. JPH203 treatment suppresses the expression of cyclin D1 (*p < 0.05) and the phosphorylation of S6 (*p < 0.05).

Inhibition of cell proliferation in the cultured ATC cells after JPH203 treatment prompted us to assess the effects of JPH203 *in vivo* using mouse xenograft models. We studied the induction of tumor growth through 8505C cell injection in athymic mice because it is the most commonly used ATC cell line. JPH203 administration intraperitoneally decreased the growth ratio of xenograft tumors (Fig. 5A, *p* < 0.05) and also reduced the tumor size (Fig. 5B). And there was no difference between body weight of the mice treated with JPH203 and body weight of the mice treated with control DMSO (p = 0.1449, Supplemental Fig. S2). Histology of the tumor between control and JPH203 treatment was similar (Fig. 5C). To confirm whether our xenograft model tumors expressed the LAT1 and 4F2hc, we performed immunohistochemistry. There were no differences in terms of the expression of LAT1 and 4F2hc proteins between JPH203-treated mice and control mice. To clarify cell growth suppression mechanism, we performed immunohistochemistry for the expression of a cell proliferation marker Ki67 in mouse tumor implanted models. Reduction of Ki67 positive cells were observed in JPH203 treatment group as compared with control group (Fig. 5C,D, p < 0.001). In addition, we found that cyclin D1 and phosphorylated S6 protein levels were lower in JPH203 treatment group than in control group (Fig. 5E,F; p < 0.05 and p < 0.05, respectively). However, LAT1 and 4F2hc expressions were not changed by JPH203 treatment. These results indicate that JPH203 suppresses tumor growth via suppression of mTOR signals as similar to our *in vitro* observations.

## Discussion

LAT1 expression seems to be one of the promising therapeutical target in various cancers. LAT1 inhibitors, JPH203, BCH, and RNAi, lead to mainly attenuate cell proliferation. Of them, several reports have provided the molecular analysis and showed the anti-tumor proliferation mechanism by western blot assay, through downregulating the phosphorylation of mTOR pathway proteins such as p70S6K, and S6, and upregulating the phosphorylation of 4EBP1^31–33^. Moreover, JPH203 activated the mitochondria-dependent apoptotic signaling pathway by upregulating pro-apoptotic factors, such as Bad, Bax, and Bak, and the active form of caspase-9, and downregulating anti-apoptotic factors, such as Bcl-2 and Bcl-xL in human osteosarcoma Saos2 cells^35^. The other groups also showed induction of the apoptosis in oral cancer YD-38 cells^36^. We demonstrated that the tumor proliferation was suppressed by inhibiting LAT1 in ATC preclinical model both *in vitro* and *in vivo*. Inhibition of LAT1 causes down regulation of mTOR signaling pathway through suppressing amino acid uptake into tumor cells. In ATC cells of 8505C, OCUT-2, and OCUT-6, the mTOR downstream signals also dramatically influenced *in vitro* model in this study (Fig. 3E–H). The suppressed mTOR signals led to the G1 cell cycle arrest by decreasing cyclin D1, CDK4, and E2F1 expressions (Fig. 4).

So far two reports provided the preclinical cancer xenograft mouse models of JPH203 administration^37,38^. JPH203 showed anti-tumor efficacy in nude mice bearing human colon cancer and cholangiocarcinoma cell xenografts with doses of 12.5 and 25 mg/kg/day. JPH203 significantly inhibited tumor growth in HT-29 and KKU-213 cell xenografts in the nude mice model in a dose-dependent manner with no toxicity. In our ATC xenograft model, JPH203 administration with a dose of 12.5 mg/day also suppressed the tumor growth through blocking downstream mTOR signaling pathway.

To the best of our knowledge, only two studies exist for targeting LAT1 in thyroid cancer^39,40^. Barollo S *et al*. reported the overexpression of the LAT1 in patients with human medullary thyroid cancer (MTC) as a part of the neuroendocrine tumors^39^. They showed that the expression of glucose transporter 1 correlated with LAT1 expression in MTC and pheochromocytoma by using RT-PCR and IHC but no prognostic analysis. They also tested the overexpression of LAT1 in MTC cell line, TT, by western blot assay. Their data imply that MTC also overexpresses LAT1, and LAT1 may be therapeutic target in MTC.

The other study, which was published very recently, focused on the role of LAT1 in ATC and papillary thyroid cancer (PTC)^40^. Although they reported similar findings to our study, the methodologies are completely different. First of all, Häfliger *et al*. analyzed LAT1 at mRNA expression level alone. On the other hand, we showed the relationship between LAT1 and histopathological differences in the protein levels by using immuohistochemical (IHC) procedures. Moreover, we evaluated the co-expressed 4F2hc protein, which is the chaperone protein of LAT1. Secondly, Häfliger *et al*. showed inactivation of mTOR pathway *in vitro*. However, they did not provide any data about cell proliferations. Our current data clearly showed the G1 cell cycle arrest through cyclin D1 and CDK4 reduction and Ki67 expression was also decreased after JPH203 treatment (Figs 3 and 4). We also showed that LAT1 knockdown by siRNA treatment attenuated mTOR signaling and cell proliferation as similar to JPH203 treatment (Supplemental Fig. S1). Finally, Häfliger *et al*. used BRAF^V600E^/PIK3CA^H1047R^ double KO mice for establishing *in vivo* ATC model. This is quite important and there are radical differences. This mouse model was well known as spontaneous ATC model based on the activated MAPK and PI3K pathway. However, it is also well recognized that the pathogenesis of human ATC involved p53 mutation with activated MAPK and PI3K pathway^41^. Our xenograft mouse model using ATC cell line 8505C that consists of BRAF, PI3K3R1/2 and p53 mutations are much popular to investigate pathogenesis of ATC. Based on these facts, our xenograft model is much appropriate for the preclinical evaluation of the effectiveness of JPH203 against ATC. Nevertheless, the difference of experimental design at the same period, their findings strongly support our conclusion. We can conclude that LAT1 inhibitors would be effective therapeutic candidates toward to ATC with strong reliability.

Recently, the novel Boron Neutron Capture Therapy (BNCT) is developed for malignant brain cancer and salivary gland carcinoma^42,43^. It is a binary radiotherapeutic modality based on the nuclear capture and fission reactions that occur when the stable isotope, boron-10, is irradiated with neutrons to produce high energy alpha particles. To deliver boron-10 into cancer cells, the low molecular weight boron-containing drugs, boronophenylalanine (BPA), currently are being used clinically. LAT1 is a well-known key protein for BNCT because LAT1 transport boronophenylalanine into cancer cells^44,45^. In present study, we found that ATC overexpresses LAT1 protein, and then our findings imply that BNCT may also be effective for ATC.

Our study also has some limitations. Firstly, we analyzed 14 ATC patients and 15 AG patients as control for histological analysis. Sample number is relatively small, and cannot show the statistical difference in survival data as shown in Fig. 1m. Secondly, we did not know the effectiveness of JPH203 for other ATC cell lines such as THJ-16, 8305C, ARO, and KTC-2, which have different gene mutations than the cells used in the current study.

A phase I clinical trial using JPH203 was completed (UMIN000016546) and a phase II clinical trial for advanced cholangiocarcinoma has just started in Japan (UMIN000034080). These trials will demonstrate promising results.

In conclusion, our study suggests that inhibition of LAT1 by JPH203 would be a novel molecular targeted therapy for one of the most fatal cancer types, ATC.

## Supplementary information

Supplementary_Figures
