# Supplementary material for: A novel therapeutic approach for anaplastic thyroid cancer through inhibition of LAT1
Source: Sci Rep. 2019 Oct 10;9:14616. doi: 10.1038/s41598-019-51144-6 (PMC6787004; doi:10.1038/s41598-019-51144-6)

**A novel therapeutic approach for anaplastic thyroid cancer through inhibition of LAT1**

Keisuke Enomoto ^1^, Fuyuki Sato ^2^, Shunji Tamagawa ^1^, Mehmet Gunduz ^1^, Naoyoshi Onoda ^3^, Shinya Uchino ^4^, Yasuteru Muragaki ^2^, Muneki Hotomi ^1^

1. Departments of Otolaryngology-Head and Neck Surgery, Wakayama Medical University, Wakayama, Japan
2. Departments of Pathology, Wakayama Medical University, Wakayama, Japan
3. Department of Breast & Endocrine Surgery, Graduate School of Medicine, Osaka City University, Osaka, Japan
4. Noguchi Thyroid Clinic and Hospital Foundation, Oita, Japan

*Correspondence to; Muneki Hotomi, Professor and Head

Department of Otolaryngology-Head and Neck Surgery, School of Medicine, Wakayama Medical University 811-1 Kimiidera, Wakayama, 641-8509, Japan

Tel: +81-73-441-0651. Fax: +81-73-446-3846;

E-mail: mhotomi@wakayama-med.ac.jp

Abbreviated title: LAT1 inhibition in anaplastic thyroid cancer

**Key terms:** JPH203, anaplastic thyroid cancer, amino acid transporter, therapy, LAT1

**Supplemental Figure S1**

Western blot analyses of LAT1 knockdown by siRNA. Representative images of LAT1 siRNA significantly decreased endogenous LAT1 protein expression in ATC cells. Expressions of 4F2hc and phosphorylated p70S6K were decreased by LAT1 knockdown using both siRNA_#1 and _#2. The cyclin D1 expression significantly decreased by LAT1 siRNA treatment in ATC cells.

**Supplemental Figure S2**

Comparison of body weight ratio of the animals according to the treatment. There is no difference between JPH203 treatment and control DMSO (p=0.1449).

Supplemental Figure 1


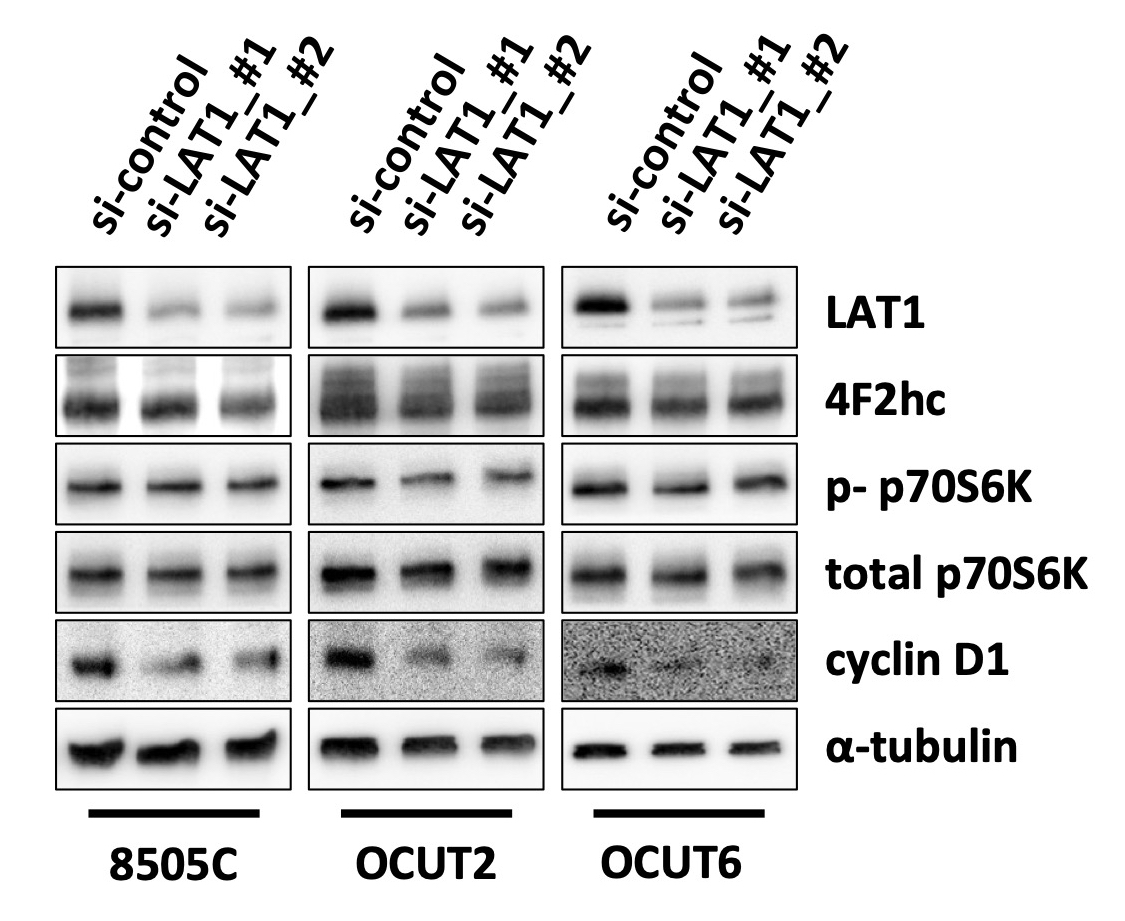


Supplemental Figure 2


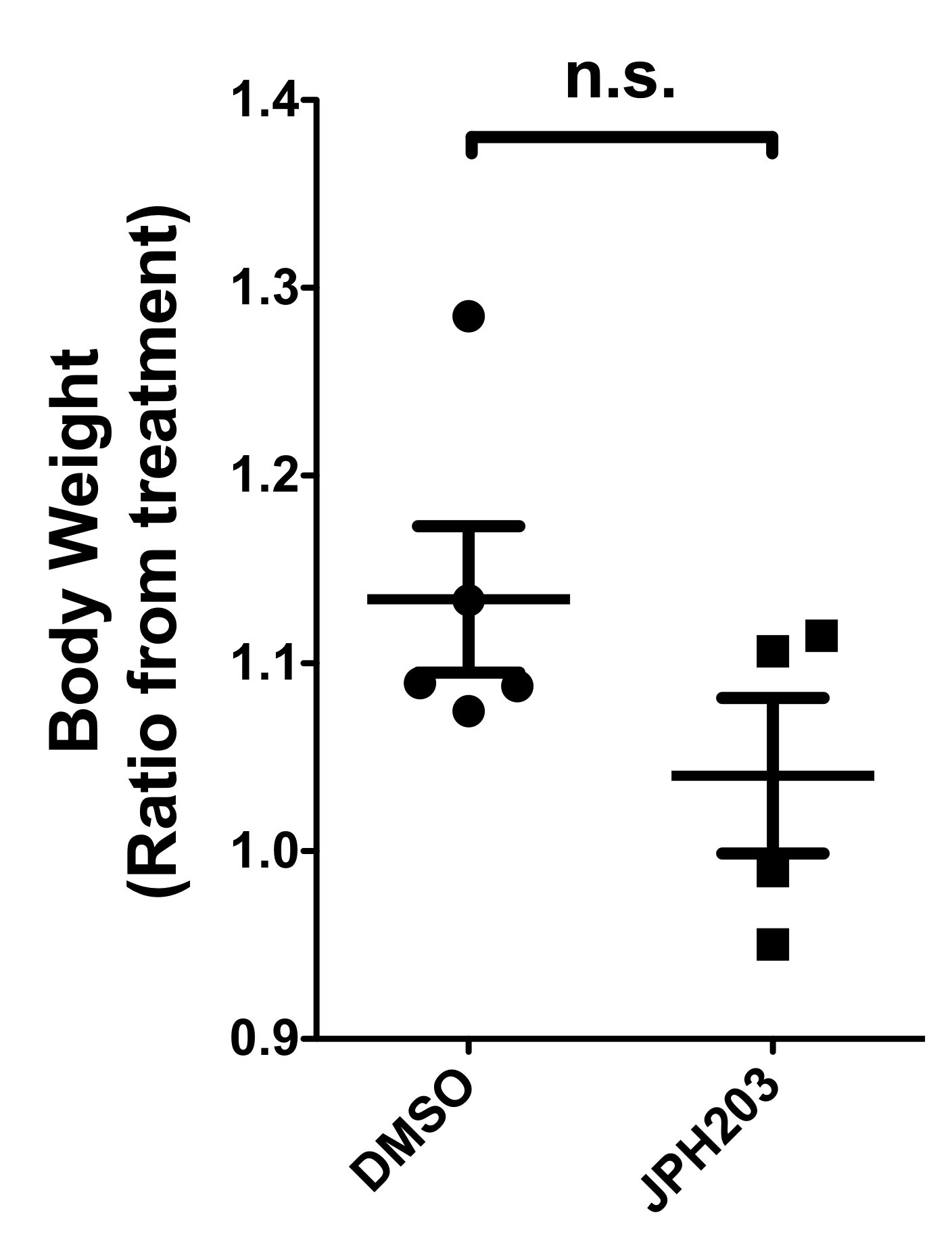

Supplement: Supplementary file 1 — Supplementary_Figures [file 41598_2019_51144_MOESM1_ESM.docx]
